# Transcriptional Profiling of Hypoxia-Regulated Non-coding RNAs in Human Primary Endothelial Cells

**DOI:** 10.3389/fcvm.2018.00159

**Published:** 2018-11-05

**Authors:** Pierre R. Moreau, Tiit Örd, Nicholas L. Downes, Henri Niskanen, Maria Bouvy-Liivrand, Einari Aavik, Seppo Ylä-Herttuala, Minna U. Kaikkonen

**Affiliations:** ^1^A. I. Virtanen Institute for Molecular Sciences, University of Eastern Finland, Kuopio, Finland; ^2^School of Medicine, University of Eastern Finland, Kuopio, Finland; ^3^Gene Therapy Unit, Kuopio University Hospital, Kuopio, Finland; ^4^Heart Center, Kuopio University Hospital, Kuopio, Finland

**Keywords:** long non-coding RNA, atherosclerosis, hypoxia, endothelial cell, super-enhancer, GRO-Seq

## Abstract

Hypoxia occurs in human atherosclerotic lesions and has multiple adverse effects on endothelial cell metabolism. Recently, key roles of long non-coding RNAs (lncRNAs) in the development of atherosclerosis have begun to emerge. In this study, we investigate the lncRNA profiles of human umbilical vein endothelial cells subjected to hypoxia using global run-on sequencing (GRO-Seq). We demonstrate that hypoxia regulates the nascent transcription of ~1800 lncRNAs. Interestingly, we uncover evidence that promoter-associated lncRNAs are more likely to be induced by hypoxia compared to enhancer-associated lncRNAs, which exhibit an equal distribution of up- and downregulated transcripts. We also demonstrate that hypoxia leads to a significant induction in the activity of super-enhancers next to transcription factors and other genes implicated in angiogenesis, cell survival and adhesion, whereas super-enhancers near several negative regulators of angiogenesis were repressed. Despite the majority of lncRNAs exhibiting low detection in RNA-Seq, a subset of lncRNAs were expressed at comparable levels to mRNAs. Among these, MALAT1, HYMAI, LOC730101, KIAA1656, and LOC339803 were found differentially expressed in human atherosclerotic lesions, compared to normal vascular tissue, and may thus serve as potential biomarkers for lesion hypoxia.

## Introduction

Recent transcriptomic analyses have established that up to 90% of the eukaryotic genome is transcribed (1). Only 2% of these transcripts encode for proteins, while the vast majority is transcribed as non-coding RNAs (ncRNAs). An increasing number of reports have discovered functional and structural roles for ncRNAs (e.g., microRNAs, small nucleolar RNAs, and small nucleolar RNAs), but despite this, the majority of them remain uncharacterized. The largest group of ncRNAs are called long ncRNAs (lncRNAs), which are defined as non-coding transcripts >200 nucleotides in length (2). LncRNAs can be further divided into promoter-associated lncRNAs and enhancer-associated lncRNAs (also called enhancer RNAs) based on epigenomic classification (3). Promoter-associated lncRNAs, like protein-coding mRNAs, are relatively stable, often spliced and polyadenylated, whereas enhancer RNAs (eRNAs) tend to lack these modifications and are generally unstable (4). Interestingly, lncRNA expression is exquisitely cell type-specific and is often perturbed in disease states (5, 6), suggesting functions in development, homeostasis and maintenance of cell identity.

Atherosclerotic vascular disease is a leading cause of morbidity and mortality in the developed world (7). A critical early step in the development of atherosclerosis is endothelial injury and the resulting endothelial dysfunction which stimulates an infiltration of leukocytes into the vessel wall (8). In the later stages of atherosclerosis, plaque endothelial cells (ECs) are subject to an adverse microenvironment characterized by hypoxia and proinflammatory stimuli, greatly affecting their function as endothelial barrier (9). To date, some instances of specific lncRNAs involved in the maintenance of EC functions, particularly angiogenesis, have been uncovered (10). For example, MALAT1, a highly abundant lncRNA, has been implicated in EC proliferation, migration and tube formation (10). Several lncRNAs have been linked to the control of hypoxia responses via the modulation of hypoxia-inducible factor 1α (HIF1α) activity in the context of tumor hypoxia (11), representing molecular mechanisms that could also be active in hypoxic atherosclerotic plaques. However, the diversity, expression dynamics and functions of lncRNAs in endothelial cells remain poorly characterized, in part due to technical limitations of standard RNA-Seq protocols as well as challenges in data analysis, compared to the profiling of protein-coding genes.

In the current study, we characterize the nascent lncRNA profiles of human primary endothelial cells subjected to hypoxia by performing GRO-Seq. We demonstrate that hypoxia has extensive genome-wide effects on the non-coding transcriptome, with hundreds of known and novel lncRNAs being differentially expressed. We further study the correlation of lncRNAs with coding gene expression and identify a subset of highly stable lncRNAs that are differentially regulated in human atherosclerotic lesions.

## Materials and methods

### Cell culture

Human umbilical vein endothelial cells (HUVECs) from 3 different donors were isolated from umbilical cords obtained from the maternity ward of the Kuopio University Hospital and used at passages 5–8. The 4th replicate represents a pool of HUVECs purchased from Gibco and used as passage 10 (potential of >16 population doublings guaranteed). The gene expression profiles of all donors correlated strongly. This work was carried out in accordance with the recommendations of the Research Ethics Committee of the Hospital District of Northern Savo, Kuopio, Finland. Informed written consent was received from all participants and the experiments were performed in accordance with the relevant guidelines and regulations.

HUVECs were maintained in endothelial cell growth medium (EGM; basal medium with SingleQuots supplements CC-4133; Lonza) on cell culture flasks coated with 10 g/ml fibronectin (Sigma, St Louis, MO, USA) and 0.05% gelatin and maintained at 37°C and 5% CO_2_. Hypoxia was induced in Ruskinn Invivo2 400 hypoxia workstation (Baker Ruskinn) in the presence of 1% O_2_ and 5% CO_2_ for 8 h. The 8 h timepoint was chosen to provide representation of early and late responses (12). Moreover, short exposure to hypoxia tends to promote cell survival and growth, while prolonged exposure to hypoxia leads to cell death (13). For adenoviral overexpression, cells were treated with AdCMV (empty vector), AdHIF1α and AdHIF2α (constitutively active forms of HIFs) (14).

### GRO-Seq and RNA-Seq

For GRO-Seq nuclei isolation, cells were treated with cycloheximide (0.1 mg/ml) for 10 min, PBS-washed and incubated in 10 ml of swelling buffer [10 mM Tris-HCl, 2 mM MgCl_2_, 3 mM CaCl_2_, and 2 U/ml SUPERase Inhibitor (Thermofisher, Waltham, MA, U.S.A.)] for 5 min on ice. Cells were scraped and pelleted for 10 min at 400 × g and resuspended in 500 μl of swelling buffer supplemented with 10% glycerol. Subsequently, 500 μl of swelling buffer supplemented with 10% glycerol and 1% Igepal was added drop by drop to the cells under gentle vortexing. Nuclei were washed twice with lysis buffer (10 ml of swelling buffer supplemented with 0.5% Igepal and 10% glycerol), and once with 1 ml of freezing buffer (50 mM Tris-HCl pH 8.3, 40% glycerol, 5 mM MgCl_2_ and 0.1 mM EDTA). Nuclei were counted manually using a Bürker chamber after Trypan Blue staining, centrifuged at 900 × g for 6 min and suspended to a concentration of 5 million nuclei per 100 μl of freezing buffer, snap-frozen in liquid nitrogen and stored −80°C until run-on reactions. For the run-on reaction, the nuclear run-on reaction buffer [NRO-RB; 496 μM KCl, 16.5 μM Tris-HCl, 8.25 μM MgCl2 and 1.65was preheated to 30°C. Then each ml of the NRO-RB was supplemented with 1.5 mM DTT, 750 μM ATP, 750 μM GTP, 4.5 μM CTP, 750 μM Br-UTP (Santa Cruz Biotechnology, Inc., Dallas, Texas, U.S.A.) and 33 μl of SUPERase Inhibitor (Thermofisher Scientific, Waltham, MA, U.S.A). Fifty microliters of the supplemented NRO-RB was added to 100 μl of nuclei samples, thoroughly mixed and incubated for 5 min at 30°C. RNA was then harvested by phenol-chloroform extraction [TRIzol LS (Thermofisher Scientific, Waltham, MA, U.S.A)].

For total RNA isolation, cells were treated with cycloheximide (0.1 mg/ml) for 10 min, PBS-washed and scraped into lysis buffer [1x Mammalian Polysome Buffer (Epicenter, Madison, Wisconsin), 1% Triton X-100, 1 mM DTT, 250 U/ml SUPERase Inhibitor, 7.1 U/ml Turbo DNase (Thermo Fisher Scientific, Waltham, Massachusetts, U.S.A.) and 0.1 mg/ml Cycloheximide] on ice. To assure complete lysis, the lysates were drawn up and expelled 4 times through a sterile 22–25 gauge needle. The cleared lysate was then treated with 10% SDS, snap-frozen in liquid nitrogen and stored −80°C.

GRO-seq libraries were subsequently prepared as previously described (15). The run-on products were treated with DNAse I according to the manufacturer's instructions (TURBO DNA-free Kit, Thermofisher, Carlsbad, CA, U.S.A.), base hydrolysed (RNA fragmentation reagent, Thermofisher, Carlsbad, CA, U.S.A.), end-repaired, and immuno-purified using anti-Br-UTP agarose beads (Santa Cruz Biotechnology, Inc., Dallas, Texas, USA).

Total RNA with size >200 nt for sequencing libraries was purified using the Zymo RNA Clean and Conc kit (Zymo Research, Irvine, California, U.S.A.) and rRNAs were removed using the Ribo-Zero Gold rRNA Removal Kit (Illumina, San Diego, CA, U.S.A.). This was followed by fragmentation (RNA fragmentation reagent, Thermofisher) and dephosphorylation.

Subsequently, both RNA-seq and GRO-seq RNA fragments were poly-A tailed (PolyA polymerase, New England Biolabs, Ipswich, MA, USA) according to manufacturer's instructions, followed by circularization and re-linearization. The cDNA templates were PCR amplified (Illumina barcoding) for 11–16 cycles. The GRO-seq libraries were size selected to be 180–300 bp in length. The RNA-seq libraries were selected to be 190–350 bp in length. The final, strand-specific libraries were quantified (Qubit dsDNA HS Assay Kit on a Qubit fluorometer, Thermofisher, Carlsbad, CA, USA) and pooled for 50 bp single-end sequencing with Illumina Hi-Seq2000 (GeneCore, EMBL Heidelberg, Germany).

### Mapping and data processing

Sequencing results were trimmed to remove 3' A-stretches originating from the library preparation and poor quality reads were filtered out (minimum 97% of bp over quality cutoff 10). Reads were aligned to the hg19 genome using bowtie allowing up to two mismatches and reporting only one alignment for each read. RNA-Seq data was mapped using STAR v2.5.4b to GRCh37/hg19 reference genome (16) with ENCODE standard options for long RNA-seq pipeline (1). Each sequencing experiment was visualized using custom tracks for the UCSC Genome Browser. R version 3.4.0 was used to filter the data, make calculations and create plots.

### Detection of long non-coding RNA

*De novo* lncRNA detection from GRO-Seq was performed using using Homer V4.9 (17) “findPeaks.pl” - algorithm with “–groseq” option. To separate lncRNAs from overlapping Refseq genes, “mergePeaks”-command was used with “-strand” option. Refseq annotated ncRNAs were further separated from protein coding genes based on “NR_” accession prefix (and further exclusion of those with “protein coding” annotation). To further divide lncRNAs to known and novel lncRNAs, the coordinates were intersected with the non-codeV5 database (18). When the overlap with database was over 70%, the lncRNA was assigned the non-coding RNA ID. SnoRNAs, ScaRNAs, and mature miRNAs were removed from the data.

### Data analysis

The differential gene expression analysis was performed for transcripts that were expressed (RPKM >0.5) in at least 3 samples among the 8 studied (*n* = 4 for normoxia and hypoxia) using EdgeR (19). Similarly, the correlation between the lncRNA expression and the closest coding gene was performed for the coding genes that were expressed (RPKM >0.5) in at least 3 samples among the 8 studied. This criteria has been defined in order to remove low counts in the libraries to improve the sensitivity and the precision of the differential genes expression (20). Moreover, this threshold was selected because, for GRO-Seq, reads are counted throughout the gene body, which represents more total reads per genes than RNA-seq (12, 15, 21, 22). Differentially expressed genes were defined as transcripts that exhibited over 2-fold change in expression compared to control and FDR < 5%. Ingenuity Pathway Analysis (IPA; QIAGEN) or DAVID 6.8 (23, 24) was used to analyse the pathways and gene ontologies enriched among the differentially regulated genes under hypoxia (25).

LncRNAs were divided to promoter- and enhancer associated transcripts by measuring the average signal of H3K4me3 and H3K4me1 calculated from the datasets GSE29611 and GSE39089 around 1 kb of transcriptional start site. A log_2_-ratio of H3K4me3 to H3K4me1 was calculated and positive ratios were assigned as promoter-associated transcripts and negative ratio as enhancer-associated (enhancer RNA) transcripts. Normal enhancers and super-enhancers (SEs) were detected from the normoxia and hypoxia datasets of public H3K27ac ChiP-Seq data (26) using the homer algorithm “findPeaks.pl” with “-style histone” and “-style super” settings, respectively. The eRNA expression was quantified for the combination of normal enhancers and super-enhancers from GRO-Seq and differential expression was determined using edgeR. SEs exhibiting FDR < 0.05 were selected for further analysis (Table S1). The SEs were detected for hypoxia and normoxia and those not overlapping were defined as gained/lost SEs upon hypoxia. To identify the stable lncRNA, we correlated the GRO-Seq and RNA-Seq data from two matching HUVEC donors. Highly stable lncRNAs were defined as a transcript that exhibited a RPKM >2 in RNA-seq. This analysis was performed separately for the two conditions. The expression of stable lncRNAs was compared to previously published microarray data (27) for the lncRNA genes that were represented by the Affymetrix HGU133 Plus2 array.

### Human sample data

Atherosclerotic segments of femoral arteries from 4 patients with primary atherosclerotic lesions (*n* = 4), 5 patients with restenotic lesions (*n* = 5) were compared with non-atherosclerotic arteries from 4 patients (*n* = 4). The samples were age matched and average age (+SD) of the patients for atherosclerotic plaques and normal mammary artery controls were 70.9(+7.3) and 67.2(+9.1) years, respectively (27). Atherosclerotic samples were collected at vascular atherectomy operations (28). The normal samples represent trimmed ends of mammary arteries isolated during cardiac bypass surgery. Total RNA was isolated, amplified, labeled and hybridized to Affymetrix HGU133 Plus2 microarrays comprising 54 675 probe sets) essentially as recommended by the manufacturer (Affymetrix, Santa Clara, CA, USA) exploiting 3′ IVT Express Kit (Affymetrix, Santa Clara, California, US) for probe preparation and GeneChip® Hybridization, Wash, and Stain Kit (Affymetrix, Santa Clara, California, US) for hybridization and detection. The analysis of gene expression followed standard procedures including Robust Multichip Average (RMA) data normalization. After RMA normalization (29) of the microarray raw data, a filtering step was applied to remove the weakest signals (intensities lower than 2 × above global background) and from initial 54,675 probe sets 17,589 were included in Significance Analysis of Microarrays (SAM); (30) to identify differentially expressed genes at False Discovery Rate <0.1. The studies were approved by Local Ethical Committee of Kuopio University Hospital under identification number 53/2011 and the subjects gave informed consent.

### Data access

The GRO-Seq data is available under NCBI Gene Expression Omnibus under accession number GSE103945. The public ChIP-Seq datasets analyzed can be found under accession numbers GSE29611 (H3K4me1: GSM733690 and H3K4me3: GSM733673 and H3K27ac: GSM733696) and GSE39089 (H3K4me1: GSM955981 and H3K4me3: GSM955983). The RNA-Seq from AdHIF1α and AdHIF2α overexpression are available under accession GSE98060. The microarray data from human femoral atherosclerotic lesions collected during atherectomy can be found under accession GSE53274 and GSE7307. The RNA-Seq data of hypoxia treated HUVECs and GRO-Seq data of adenovirally overexpressed constitutively active HIF1α and HIF2α have been submitted to under accession number GSE118530.

## Results

### Hypoxia regulates the expression of hundreds of coding and non-coding RNAs on transcriptional level

**Figure 1:**
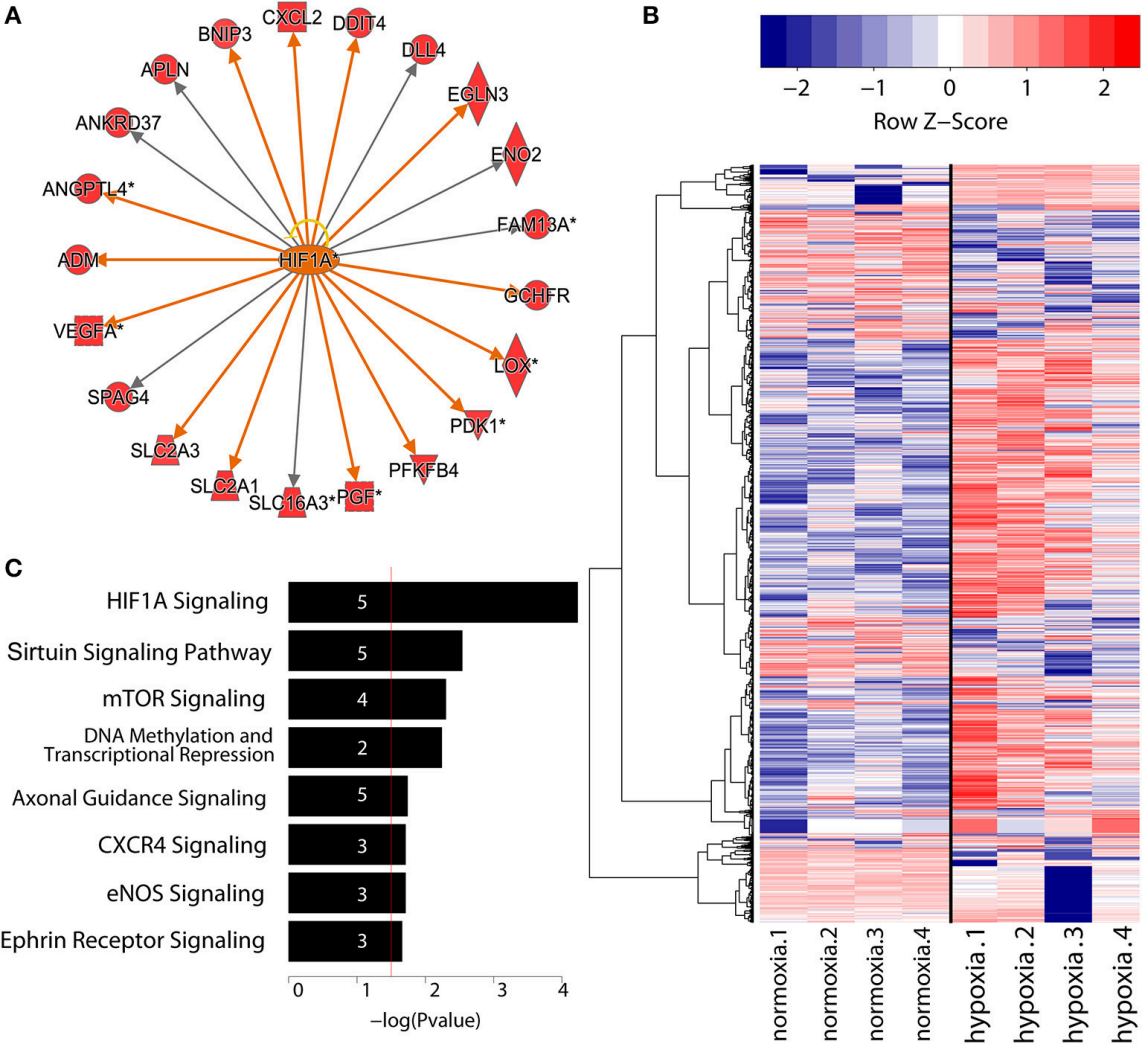
**(A)** Identification of top upstream regulators responsible for gene expression changes during EC differentiation confirmed the central role of HIF1α.Threshold was defined as FDR < 0.05 and log_2_(FC) > 1.5. Activation of downstream genes by HIF1α is indicated with orange arrows, whereas gray arrows signify unknown direction of effect. Yellow arrow indicates inconsistent findings with the state of downstream molecule. Diamond shape represents enzyme, square shape: cytokine, dotted square shape: growth factor, triangle shape: kinase, trapezoid shape: transporter, and circle shape: other. Network graph obtained using IPA (QIAGEN). **(B)** Heatmap showing the expression values in log_2_(RPKM) of differentially regulated lncRNAs (FDR < 5%). Throughout the paper, the normalization is in RPKM. Rows are hierarchically clustered using Ward's least absolute error with Minkowski distance. And colors are row scaled. **(C)** Gene ontology analysis of lncRNAs under hypoxia condition generated with IPA (QIAGEN). Numbers in the bars indicate the amount of genes included in the pathways.

To study the nascent transcriptomes of ncRNAs in primary human endothelial cells, we performed GRO-Seq on HUVECs subjected to 8 h of hypoxia (1% oxygen). Altogether, we identified 33,508 transcripts above the expression threshold of 0.5 RPKM in at least 3 samples. Among these, 468 protein coding transcripts were found upregulated and 57 downregulated at least 2-fold (with false discovery rate below 5%) in response to hypoxia (Table S2A). We further analyzed these genes for gene ontology and upstream regulators using Ingenuity Pathway Analysis. As expected, HIF1α signaling and glucose metabolism were among the top canonical pathways enriched (Table S3) and HIF1α was identified as the top upstream regulator of the differentially expressed genes (Figure 1A). This confirms that a significant fraction of hypoxia-regulated genes are regulated on a transcriptional level.

In addition to protein-coding genes, we identified 1,763 differentially regulated lncRNAs in our analysis (applying a false discovery rate threshold of 5%), with 544 upregulated and 350 downregulated more than 2-fold. (Table S2B, Figure 1B). Eight hundred and eight of these lncRNAs corresponded to known lncRNAs (RefSeq or NONCODE) (Figure 1C) and 955 were novel, previously uncharacterized lncRNAs. Altogether, this demonstrates that hypoxia regulates a markedly larger set of non-coding RNAs than protein coding transcripts.

### Promoter-associated lncRNAs are enriched for upregulated transcripts

**Figure 2:**
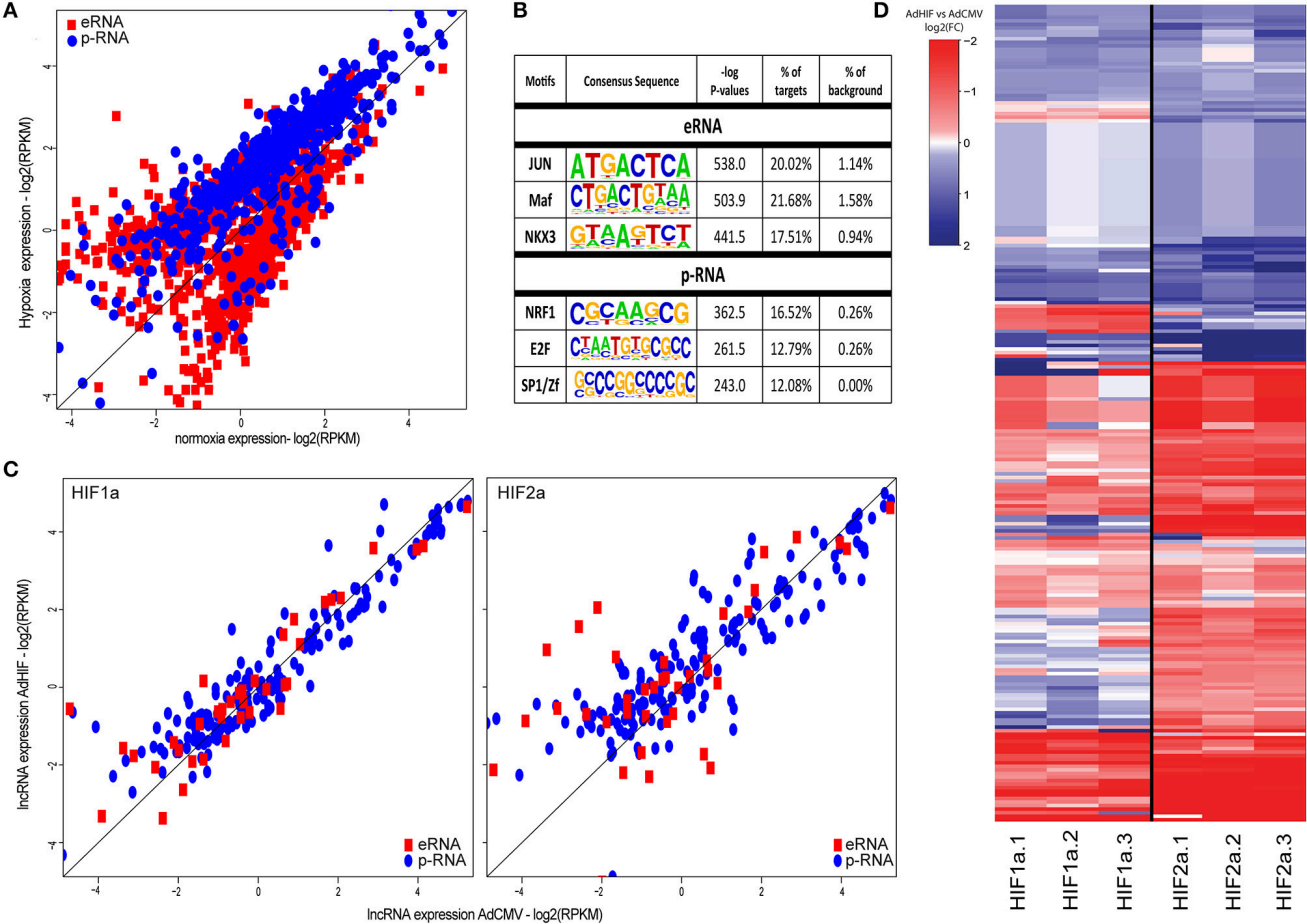
**(A)** Scatter plot of the expression values (log_2_ RPKM) of differentially expressed lncRNAs in response to hypoxia (FDR < 5%). Red squares represent lncRNAs exhibiting high H3K4me1 signal (eRNAs), blue dots represent lncRNAs exhibiting high H3K4me3 signal (p-RNAs). **(B)** DNA-binding motif enrichment analysis at the transcription start sites of the two lncRNA categories (±500 bp). All differentially regulated lncRNAs were used as background. **(C)** Scatter plot showing the nascent lncRNA expression (log_2_ RPKM of GRO-Seq signal) after adenoviral overexpression of constitutively active forms of HIF1α or HIF2α compared to adenovirus without transgene (AdCMV). Red squares represent eRNAs, blue dots represent p-RNAs. **(D)** Heatmap showing the log_2_(FC) of the lncRNAs after adenoviral overexpression of HIF1α or HIF2α compared to adenoviral overexpression of CMV based on RNA-Seq. Rows are hierarchically clustered using Ward's least absolute error with Euclidian distance.

To further classify the lncRNAs based on their epigenomic features, we divided the transcripts to promoter- and enhancer-associated lncRNAs according to higher enrichment for H3K4me3 and H3K4me1 histone marks, respectively (31). Altogether, we were able to define 707 promoter-associated lncRNAs (p-lncRNA) and 1056 enhancer RNAs (eRNAs) (Figure 2A). Surprisingly, majority (90%) of the differentially regulated p-lncRNAs were induced upon hypoxia stimulus, in contrast to eRNAs that showed an approximately equal distribution of expression change directions (Figure 2A). To identify possible mechanisms underlying the differences in expression profiles of different types of lncRNAs, we performed *de novo* motif analysis near their transcription start sites. Clear differences were seen among the top transcription factor motifs enriched, with JUN/AP-1, MAF and NKX3 transcription factor motifs being enriched at the start sites of eRNAs, whereas NRF1, E2F, and SP1 were enriched at the promoters of p-lncRNAs (Figure 2B).

The transcriptional response to hypoxia is known to be controlled by the two master regulators, HIF1α and HIF2α, which are able to collaborate with distinct transcription factors (14, 32). To see whether differences in the usage of different HIF α-subunits contributes to differential activation of lncRNA subtypes, we further assessed the regulation of p-lncRNAs and eRNAs by overexpressing the constitutively active HIF1α and HIF2α proteins in HUVECs for 48 h. Western blot analysis of the HUVEC lysates demonstrated high expression of HIF1α and HIF2α protein levels relative to the AdCMV-transduced control cells (Figure S1). The GRO-Seq analysis demonstrated that HIF2α overexpression led to more induced p-lncRNAs (54%−140/260) compared to HIF1α (40%−104/260) (Figure 2C). The higher induction of lncRNA expression by HIF2α was further confirmed using RNA-Seq (Figure 2D).

### Hypoxia response leads to significant regulation of super-enhancer activity

**Figure 3:**
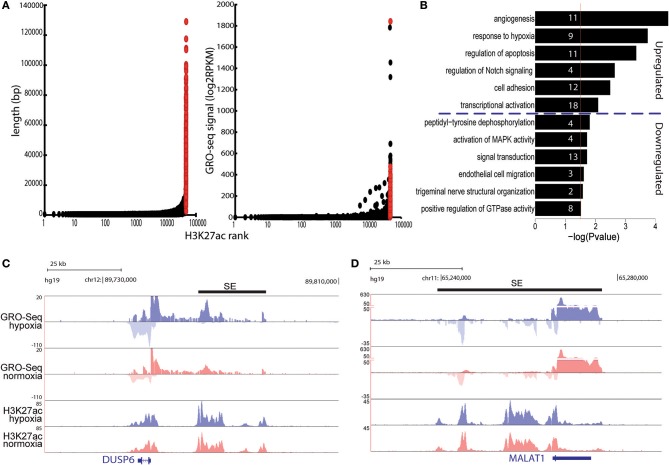
**(A)** Left: total length of super-enhancers (red) and normal enhancers (black) ranked by increasing H3K27ac signal under hypoxia. Right: total GRO-Seq signal (count per million) for super-enhancers and normal enhancers using the H3K27ac based ranking. **(B)** Gene ontology analysis of the differentially expressed genes located <100 kb from induced and repressed superenhancers. Gene ontology was performed with DAVID. Numbers in the bars indicate the amount of genes included in the pathways. **(C,D)** UCSC genome browser shot images of DUSP6 **(C)** and MALAT1 **(D)** under hypoxia (blue) and normoxia (red). Normalized tag counts are shown for GRO-Seq and ChIP-Seq. Black bars represent the super-enhancer position.

Recent studies have suggested that the expression of cell type-specific genes is controlled through clusters of enhancers called super-enhancers (33). There is also accumulating evidence that these enhancer-dense genomic regions play key roles in cellular response to stimulus (34, 35). To study how hypoxia affects SEs, we used H3K27ac profiles from HUVECs subjected to normoxia and hypoxia for 24 h. Altogether, 1058 and 799 SEs were identified under normoxia and hypoxia conditions, respectively, which were highly concordant with the number of published basal HUVEC SEs (33). As the ChIP-Seq data lacked sufficient replicates and GRO-Seq data can also serve as a reliable indicator of enhancer activity, we used our GRO-Seq data from 4 independent replicates to identify which of these SEs are hypoxia-regulated. The results show that hypoxia led to significant regulation of 573 SEs (Table S1, Figure 3A) by inducing the expression of 358 SEs and repressing the activity of 215. Interestingly, the induced SEs were found nearby differentially expressed coding genes related angiogenesis (e.g., PGF, MMP2, DLL4, EGFL7, and TGFB1), regulation of apoptosis (e.g., NOTCH1, SOX4, ANKRD1, and DUSP6), cell adhesion (e.g., CD34, CDH5, VWF, and CTNNB1), and transcriptional activation (e.g., MEF2A, SOX4, EGLN1, MAFK, ASH2L, and TCEA2), whereas the downregulated SEs were close to regulators of peptidyl-tyrosine dephosphorylation (e.g., DUSP6, PTP4A2, PTPN14, and PTPN1) and genes involved in signal transduction (e.g., IL15, IL15RA, and MAP2K6) and GTPase activity (e.g., ARHGAP7/18/24/29; Table S4, Figures 3B,C). Notably, for 20% of SEs the closest RefSeq gene was a lncRNA, as exemplified by MALAT1 (Figure 3D) and LUCAT1.

### LncRNAs expression is co-regulated with nearby protein-Coding genes and mostly represented by unstable RNAs

**Figure 4:**
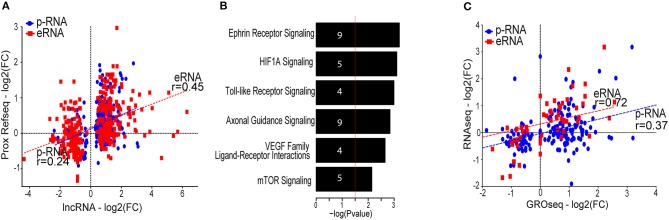
**(A)** Scatter plot showing the correlation between the lncRNA and the proximal (<100 kb) protein coding genes. Only proximal coding genes exhibiting differential gene expression (FDR value below 5% and a fold change threshold of ±1.5) are displayed. Correlation calculated using Spearman correlation. **(B)** Gene ontology analysis of the differentially expressed proximal coding genes generated with IPA (QIAGEN). Numbers in the bars indicate the amount of genes included in the pathways. **(C)** Scatter plot showing the fold change (log_2_) of RNA-Seq compared to the fold change (log_2_) of GRO-Seq of the differentially expressed lncRNAs. Transcripts exhibiting low expression (RPKM < 0.5 in <2 samples) were removed from the analysis. Correlation calculated using Spearman correlation.

We next correlated the fold change of lncRNAs and their nearby coding genes in response to hypoxia. The results demonstrated a good correlation with the coding gene that was stronger for eRNAs than for p-lncRNAs (Figure 4A). This supports the concept that coding genes could be co-regulated through sharing of regulatory motifs or cis-regulation mediated by lncRNAs (36).

To infer functions of the identified lncRNAs, we applied the guilt-by-association principle: if lncRNA shows an expression profile that correlates with nearby protein-coding genes involved in a given function, the lncRNA may be involved in the same function. Gene ontology analysis indicates that genes expressed in close proximity to hypoxia-regulated lncRNAs are mostly involved in angiogenesis and cell migration, as exemplified by the induction of pathways such as ephrin signaling, HIF1α and the VEGF family ligand-receptor interactions pathway (Figure 4B). This analysis thus predicts that lncRNAs could be important in hypoxia-mediated changes in endothelial cell signaling and function.

LncRNAs could also mediate *trans*-effects, which likely require the lncRNA transcript to be abundant or long-lived (37–39). To identify this subgroup of stable lncRNAs, we compared the GRO-Seq expression levels to RNA-Seq data from matching HUVEC donors. Here, RNA-Seq is expected to reflect the stable lncRNA pool within cell, whereas GRO-Seq is able to display all nascent transcripts irrespective of their half-life. Altogether, 13% of the lncRNAs seen in GRO-Seq were detected using RNA-Seq, and majority of these were associated with promoter-signature (among the 236 detected lncRNAs, 180 were promoter-associated lncRNAs and 56 were enhancer-associated lncRNAs). This was in contrast to protein-coding genes, for which 84% of GRO-Seq-detected genes exhibited detectable expression in RNA-Seq as well (data not shown). The low fraction of lncRNAs detected using RNA-Seq highlights the advantage of GRO-Seq in capturing a large amount of unstable lncRNAs that would otherwise be missed. Interestingly, the hypoxia regulation of eRNAs (fold change in GRO-Seq) exhibited a higher correlation with RNA-Seq than that of p-lncRNAs, suggesting that larger fraction of stable p-lncRNAs could be further regulated at the post-transcriptional level (Figure 4C, Figure S2). Supporting this, similarly to mRNAs, the p-lncRNAs have been shown to be targeted by cytoplasmic miRNAs and even act as competing endogenous RNAs (40), whereas the eRNAs are less likely to leave the nucleus.

### Regulation of lncRNAs in human atherosclerotic lesions

**Figure 5:**
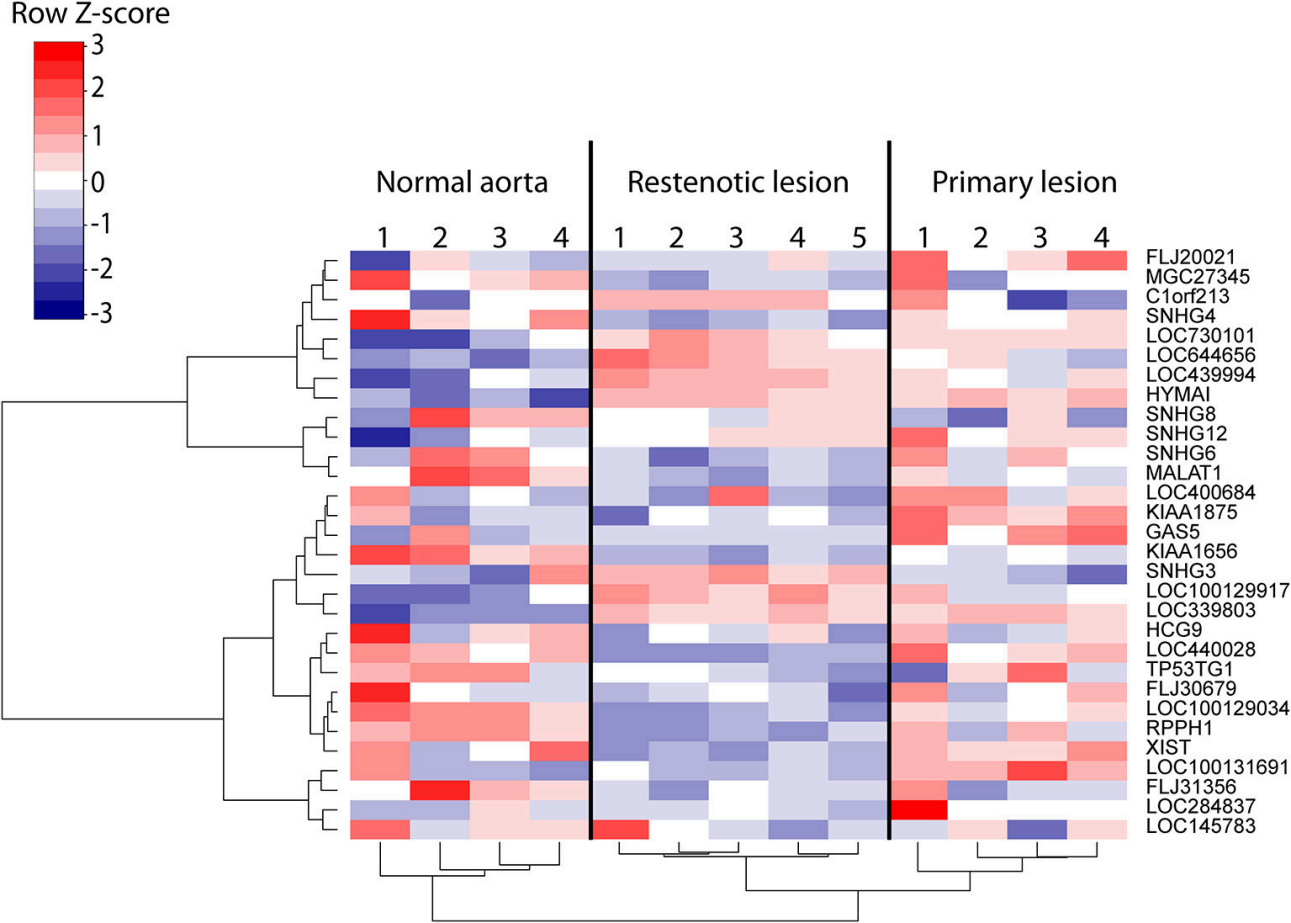
Heatmap of the differentially regulated lncRNAs (FDR < 5%) in 9 samples of human atherosclerotic lesions compared to 4 control regions from non-affected mammary arteries (27). Rows and columns clustered using Ward's least absolute error with Manhattan distance.

We subsequently investigated whether the expression of the stable lncRNAs is altered during atherosclerosis. To this end, the atherosclerotic segments of femoral arteries from 4 patients with primary atherosclerotic lesions and 5 patients with restenotic lesions were compared with non-atherosclerotic mammary arteries from 4 patients (27). Altogether 30 hypoxia-regulated lncRNAs from our HUVEC analysis were represented by the probe sets of the microarray and found to be differentially regulated in the atherosclerotic samples compared to the control tissue. This included for example the well-known lncRNA MALAT1 (Figure 5) and the lncRNA HYMAI, which has been studied in relation to diabetes (41, 42) but not previously reported in association with atherosclerosis or hypoxia. Several lncRNAs with the notable ability to distinguish between healthy and diseased vascular tissue, such as LOC730101, KIAA1656, and LOC339803, remain poorly studied. However, LOC730101 has been implicated in the activation of Wnt/β-catenin signaling (43), a pathway that is critical for endothelial cell proliferation and migration (44). Notably, restenosis samples following the intravascular surgery of the primary lesion formed a separate cluster from the primary lesions, demonstrating further altered lncRNA profiles during intimal hyperplasia and arterial remodeling in the treated arteries. Altogether, our results indicate significant regulation of lncRNA expression in human atherosclerotic lesions which could contribute to atherosclerotic lesion progression or serve as indicators of lesion hypoxia.

## Discussion

Currently, over 200,000 unique lncRNAs have been discovered across 50 human tissues or cell lines (45, 46). Here, we expand that list of lncRNAs and contribute to the characterization of hypoxia-regulated lncRNAs in human primary endothelial cells. Tissue hypoxia occurs as a part of diseases such as coronary artery disease and cerebrovascular disease, and cells have evolved complex response programs to try to manage and resolve oxygen deficiency (9). Being the innermost layer of cells in the blood vessel, endothelial cells are directly exposed to alterations in blood composition, such as hypoxia, and play a key role in maintaining vascular homeostasis. In particular, the vasculature responds to hypoxic stress by activating angiogenesis and endothelial cell proliferation and migration (9).

By carrying out GRO-Seq, an RNA profiling technique that targets ongoing transcription and is indifferent to transcript poly-adenylation, we were able to detect the differential expression of nearly 1800 lncRNAs in endothelial cells in response to hypoxia. Notably, the number of regulated lncRNAs outnumbered that of differentially expressed protein-coding genes under the same conditions (around 500). The most highly upregulated lncRNAs included many that were novel, as well as several lncRNAs described to carry out crucial functions in other types of disease or stress conditions. For example, hypoxia resulted in the marked upregulation of LUCAT1, an anti-apoptotic lncRNA recently found to affect DNA methylation by regulating DNMT1 (47), and RASSF1-AS1, which is known to downregulate the level of pro-apoptotic regulator/tumor suppressor RASSF1 (48). Induction was also seen for IDH1-AS1, a lncRNA involved in the control of energy metabolism (49). The induction of these lncRNAs in endothelial cells could promote cell survival and proliferation to facilitate vascular reorganization upon hypoxia.

By integrating GRO-Seq and ChIP-Seq data, we found that a large fraction of H3K4me1-supported lncRNAs (60%) originate from enhancers. This result is in good agreement with the recent FANTOM5 data obtained using cap analysis of gene expression (CAGE), which demonstrated that 68% of lncRNAs originate from enhancers rather than from promoters (3). We acknowledge that the classification of lncRNAs based in epigenetic marks is far from perfect with emerging evidence of highly active enhancers displaying H3K4me3-promoter mark (50) and promoters serving as enhancers (51, 52). However, this classification allowed us to distinguish that promoter associated lncRNAs and mRNAs are more prone to gene activation in response to hypoxia, compared to enhancer-associated lncRNAs that displayed equal level of transcriptional activation and repression. Our results demonstrate that promoter activation is more evident upon HIF2α activation but also likely involves the differential usage of collaborating transcription factors. To this end, the most highly enriched transcription factor motifs for promoter- and enhancer-associated lncRNAs were clearly distinct. Regions near eRNAs exhibited strongest enrichments for AP-1/Jun and NKX3, factors previously implicated in the control of vascularization (53, 54), and MAFA, a known regulator of energy homeostasis that can act both as activator and repressor of transcription (55). In line with their genomic origin, p-lncRNAs revealed enrichment for motifs known to be promoter-proximal, such as binding motifs for Sp1 and NRF1 (56, 57). Moreover, the most highly overrepresented motifs found near p-lncRNAs (NRF1, E2F, and Sp1) all bind factors associated with cell growth and proliferation (58–60), in line with observations that short-term and/or moderate hypoxia stimulates endothelial cell proliferation (13). We have also recently shown that HIF2α contributes more to the regulation of proliferation-related genes in endothelial cells compared to HIF1α (14). Altogether, our data suggests that HIF2α might collaborate with other transcription factors to mediate preferential activation of p-lncRNAs. Future studies are needed to disentangle the complex mechanisms of transcriptional regulation and the collaborative networks of transcription factors in response to hypoxia.

Our data shows that hypoxia leads to a remodeling of the super-enhancer landscape, with induction and repression of SE activity in the vicinity of genes known to be critical for endothelial cell function. To this end, the SE activity was significantly regulated next to several genes encoding for angiogenic factors, such as EGFL7 (61), DLL4 (62), MMP2 (63), and TGFB1 (62). In accordance with this, SE activation was also seen near several transcriptional regulators, for example MEF2, which is known be a transcriptional effector of VEGF responsible for activating DLL4 expression to drive sprouting angiogenesis (64). On the other hand, decreased SE activity was detected near several genes encoding for Rho GTPase-activating proteins, notably including the known angiogenesis inhibitors ARHGAP7 (DLC1) (65) and ARHGAP18 (66), and near several protein tyrosine phosphatase genes, including PTPN14 and PTPN1 (PTP1B), which have been reported to negatively modulate angiogenesis by regulating VEGF receptor signaling (67, 68). Similarly to protein coding genes, the regulation of SE activity was also evident next to functionally notable lncRNAs, such as the angiogenic lncRNA MALAT1 (10, 69). Thus, profiling enhancer activity by GRO-Seq yields valuable candidate regions for genetic elements that mediate endothelial cell functional responses.

Taken together, our data indicates that dynamic changes in the SEs likely serve an important role in orchestrating the growth of new blood vessels in response to hypoxia. Importantly, the modification of endothelial cell responses through the alteration of SE activity also represents a possible therapeutic approach. For example, promising results have been obtained in modulating endothelial cell inflammatory responses by targeting SEs through the inhibition of bromodomain and extra-terminal domain (BET) factors, a family of transcriptional co-activators and elongation factors (34). Crucially, SEs seem to be more sensitive to BET perturbation than typical enhancers, and the expression of their nearby genes seems to be preferentially affected (70), making BET perturbation a good therapeutic approach.

To evaluate the expression of hypoxia-responsive lncRNAs *in vivo*, we explored the correlation between lncRNA regulation in endothelial cells and human advanced atherosclerotic lesions. Other studies have previously reported on the importance of the lncRNA expression in cardiovascular development and pathophysiology (71–73), and it has been suggested that lncRNAs could serve as circulating biomarkers of endothelial injury associated with vascular diseases (10, 74). Considering the differential expression of the lncRNAs alongside the expression of coding genes and other markers could provide improved insight into the disease state of the patients, for example by helping pinpoint the activation of different types of cellular stress responses. Our results revealed several lncRNAs, such as HYMAI, LOC730101, KIAA1656, and LOC339803 that are transcriptionally induced in endothelial cells during hypoxia and overexpressed in human atherosclerotic plaques compared to control artery samples. Little is currently known about these lncRNAs; however, LOC730101 has previously been implicated in cell survival during bioenergetic stress (75). Moreover, we show that MALAT1 is downregulated in restenotic vasculature and atherosclerotic lesions. MALAT1 overexpression was first described in cancer (76) and in response to 24 h hypoxic stimulus (77, 78). However, there is increasing evidence that the direction of MALAT1 deregulation is dependent on the disease context (79) and recently MALAT1 was described to be downregulated in tissue samples from atherosclerotic coronary artery plaques (80). This is in line with our findings, were MALAT1 was downregulated in both primary and restenotic lesions. This also suggests that other factors than hypoxia could contribute to altered expression of MALAT1 in tissues and the regulation of MALAT1 is highly tissue and cell-type specific. Future studies testing the applicability of these lncRNAs for early detection of disease from blood, as well as detailed investigations into their expression profiles and regulatory mechanisms, are envisioned.

In conclusion, we report a nascent transcriptome atlas of human hypoxia-sensitive lncRNAs in primary endothelial cells and identify several lncRNAs with potential for usage as indicators of endothelial hypoxia in human atherosclerotic lesions or other vascular diseases. Future studies are expected to provide more detailed knowledge of the functional roles of the identified lncRNAs in atherogenic processes, thereby potentially helping to establish lncRNAs as future therapeutic or prognostic targets.

## Author contributions

PRM, MUK, and SY-H conceived and designed the experiments. PRM, MUK, Tö, NLD, HN, EA, and SY-H performed the data analysis. MB-L, NLD, EA, and HN acquisition of data. PRM, Tö, MUK, and MB-L drafting the manuscript. All the authors interpreted the data and revised the manuscript for content and accuracy.

### Conflict of interest statement

The authors declare that the research was conducted in the absence of any commercial or financial relationships that could be construed as a potential conflict of interest.

The reviewer UR and handling editor declared their shared affiliation at the time of the review.

## Abbreviations

EC: endothelial cell
GRO-Seq: global run-on sequencing
HIF: hypoxia-inducible factor
HUVEC: human umbilical vein endothelial cell
lncRNA: long non-coding RNA
p-lncRNA: promoter-associated lncRNA
eRNA: enhancer-associated lncRNA
SE: super-enhancer.
