# Supplementary figures and images for: Transcriptional Profiling of Hypoxia-Regulated Non-coding RNAs in Human Primary Endothelial Cells
Source: Front Cardiovasc Med. 2018 Nov 5;5:159. doi: 10.3389/fcvm.2018.00159 (PMC6230589; doi:10.3389/fcvm.2018.00159)

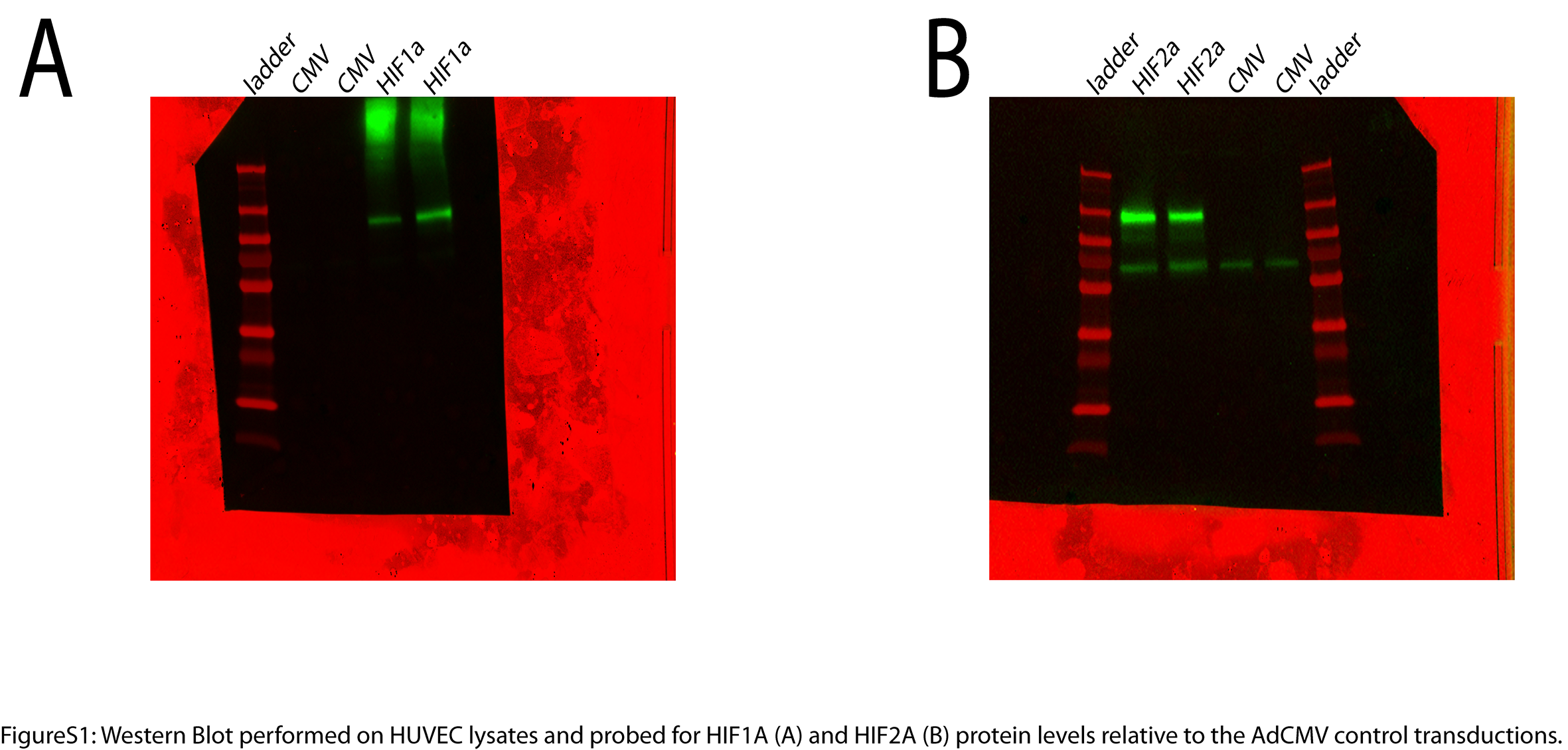

Supplement: Supplementary file 2 [file Image_1.TIF]

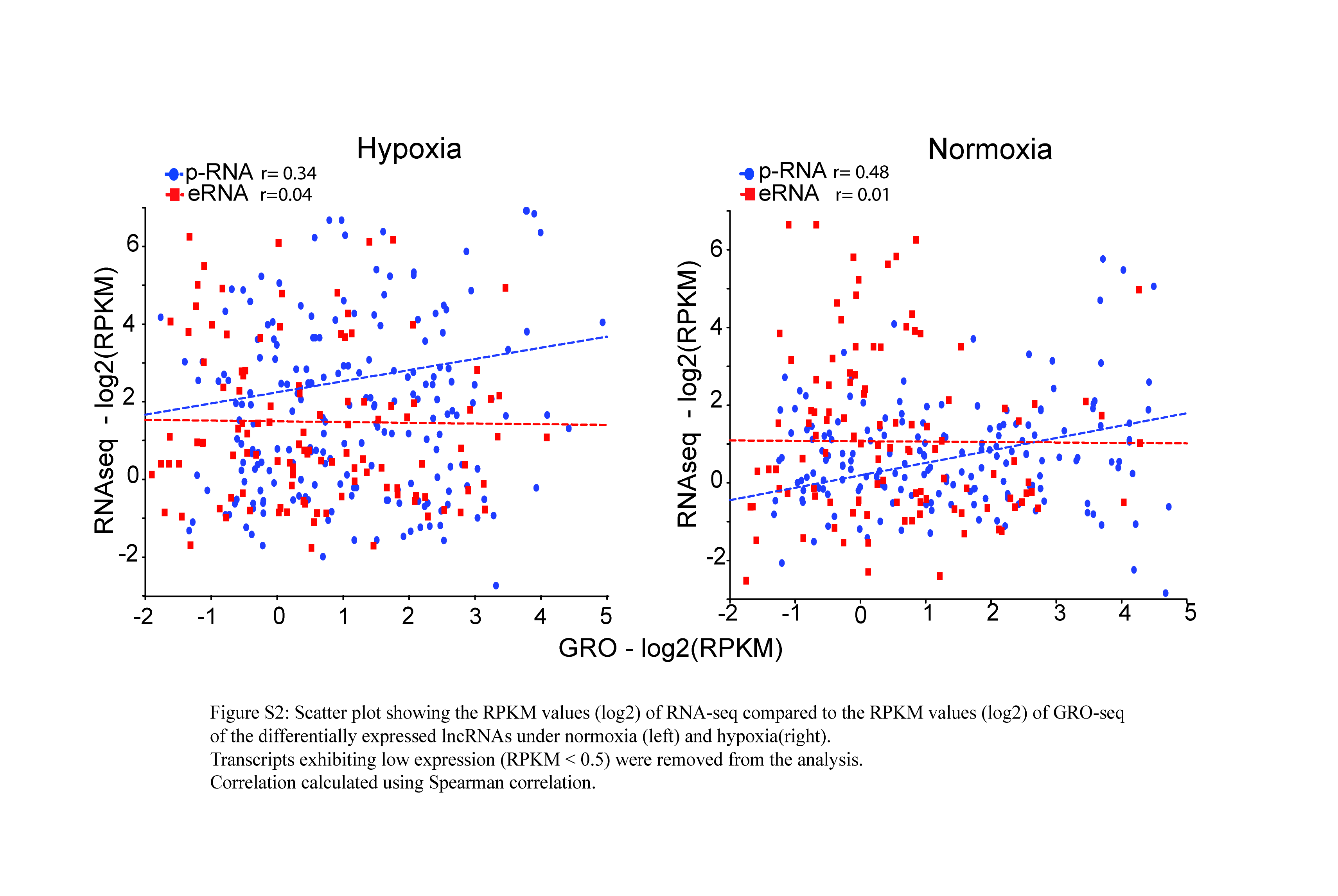

Supplement: Supplementary file 3 [file Image_2.TIF]
